# Design, Synthesis, and Biological Evaluation of New 2-Phenyl-4*H*-chromen-4-one Derivatives as Selective Cyclooxygenase-2 Inhibitors

**DOI:** 10.3797/scipharm.1407-20

**Published:** 2014-09-15

**Authors:** Afshin Zarghi, Samaneh Kakhki

**Affiliations:** Department of Pharmaceutical Chemistry, School of Pharmacy, Shahid Beheshti University of Medical Sciences, Tehran, Iran

**Keywords:** Chromene derivatives, Docking studies, COX-2 inhibitory activity

## Abstract

In order to develop new selective COX-2 inhibitors, a new series of 2-phenyl-4*H*-chromen-4-one derivatives possessing a methylsulfonyl pharmacophore group at the *para* position of the C-4 phenyl ring were designed, synthesized, and evaluated for cyclooxygenase-2 inhibitory activity. *In vitro* COX-1/COX-2 isozyme inhibition structure-activity studies identified 3-(benzyloxy)-2-[4-(methylsulfonyl)phenyl]-4*H*-chromen-4-one (**5d**) as a potent COX-2 inhibitor (IC_50_ = 0.07 μM) with a high COX-2 selectivity index (SI = 287.1) comparable to the reference drug celecoxib (COX-2 IC_50_ = 0.06 μM; COX-2 SI = 405). A molecular modeling study where 3-(benzyloxy)-2-[4-(methylsulfonyl)phenyl]-4*H*-chromen-4-one (**5d**) was docked into the active site of COX-2 showed that the *p*-MeSO_2_ substituent on the C-4 phenyl ring was well-oriented in the vicinity of the COX-2 secondary pocket (Arg^513^, Val^523^, and His^90^) and the carbonyl group of the chromene ring could interact with Ser^530^. The structure-activity data acquired indicated that the nature and size of the substituent on the C-3 chromene scaffold are important for COX-2 inhibitory activity. Our results also indicated that the chromene moiety constitutes a suitable template to design new COX-2 inhibitors.

## Introduction

Non-steroidal anti-inflammatory drugs (NSAIDs) are an important class of drugs that provide analgesic, antipyretic, and anti-inflammatory effects. Their pharmacological effects arise from the non-selective inhibition of the cyclooxygenase (COX) enzyme, a key enzyme in the arachidonic acid pathway that leads to the biosynthesis of prostaglandins. However, the applications of NSAIDs are limited due to gastric ulceration, bleeding, and renal function suppression [1–4]. It is well-established that there are at least two COX isoenzymes, COX-1 and COX-2. COX-1 is constitutively expressed in most tissues such as the gastric tract (GI) to produce cytoprotective prostaglandins to maintain the physiologic function, whereas COX-2 as an inducible isoform, is occasionally involved in pathologic processes [5, 6]. In addition, recent studies also showed the overexpression of COX-2 in both malignant lesions [7–10] and neurodegenerative diseases such as Parkinson’s [11] and Alzheimer’s disease [12–14]. Thus, the selective inhibition of COX-2 over COX-1 is useful for the treatment of inflammation and inflammation-associated disorders when compared with NSAIDs. COXIBs such as rofecoxib and celecoxib are the most important class of selective COX inhibitors. COXIBs belong to a class of compounds that frequently possess two vicinal diaryl moieties attached to a central hetero- or carbocyclic ring. A suitable substituent such as methanesulfonyl (MeSO_2_) or sulfonamide (SO_2_NH_2_) at the *para* position of one of the phenyl rings frequently confers selective COX-2 inhibitory activity. However, an increased risk of myocardial infarction and cardiovascular thrombotic events associated with the use of some selective COX-2 inhibitors has been observed. For this reason, rofecoxib as a well-known COX-2 inhibitor was withdrawn from the market due to cardiovascular side effects [15]. In order to obtain new COX-2 inhibitors with better safety and more efficacy, there is still a need to do new investigations on the development of new scaffolds as COX-2 inhibitors. In this regard, we reported several investigations describing the design, synthesis, and COX inhibitory activities of a novel class of compounds possessing an acyclic 1,3-diarylprop-2-en-1-one structural template [16]. For example, (2*E*)-3-(4-methylphenyl)-1-[4-(methylsulfonyl)phenyl]prop-2-en-1-one (see Structure **A**) was identified as a potent and selective COX-2 inhibitor. Recently, we also reported several investigations describing the design, synthesis, and molecular modeling studies for a group of 2-phenyl-4-carboxylquinolines possessing a methylsulfonyl COX-2 pharmacophore at the *para* position of the C-2 phenyl ring in conjunction with various substituents at the C-7 and C-8 quinoline ring [17]. In this group, 2-(4-(methylsulfonyl)phenyl)quinoline-4-carboxylic acid (see Structure **B**), having lipophilic substituents at the C-7 and C-8 positions, exhibited higher selectivity for COX-2 inhibition than the reference drug celecoxib. As part of our continuing program to discover selective COX-2 inhibitors, we now describe the synthesis and biological evaluation of a group of 2-phenyl-4*H*-chromen-4-one derivatives possessing a methylsulfonyl pharmacophore at the *para* position of the C-4 phenyl ring in conjunction with various substitutes at the C-3 chromene moiety. In this study, the acyclic 1,3-diarylprop-2-en-1-one structural template (**A**) as a lead compound was converted to a cyclic ring (chromene) similar to 2-aryl quinolines (**B**) to fulfill the goals of better COX-2 inhibitory potency and selectivity.

**Fig. 1:**
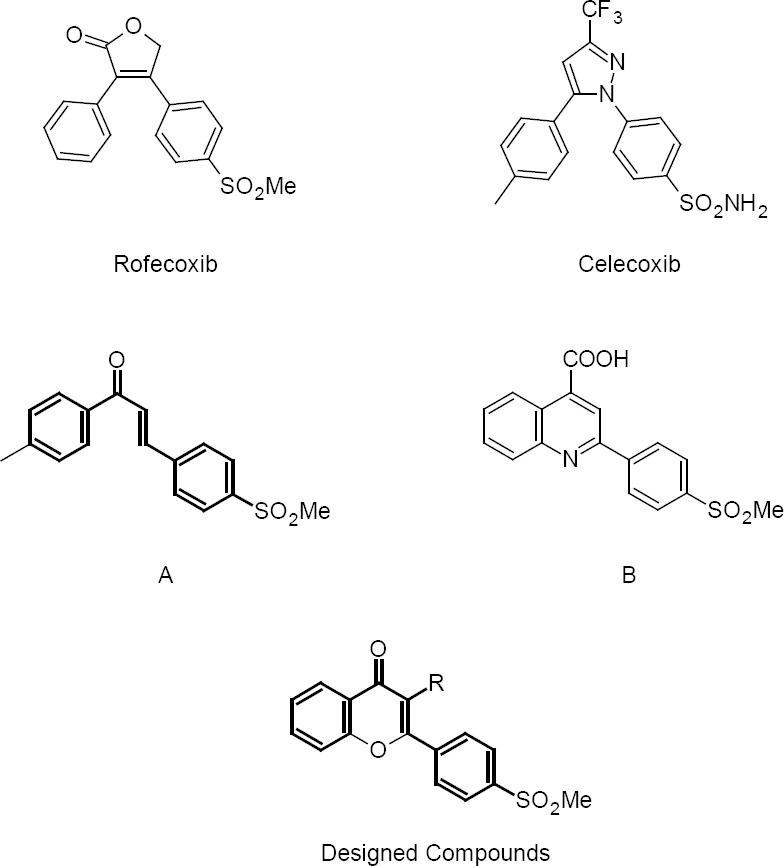
Some representative examples of selective COX-2 inhibitors and our designed compounds

## Results and Discussion

The synthetic strategies used to prepare the target 2-[4-(methylsulfonyl)phenyl]-4*H*-chromen-4-one derivatives **3–6** are illustrated in Scheme 1 [18]. Using aldol condensation, the desired chalcone **1** was obtained through the reaction between 4-(methylthio)-benzaldehyde and 2-hydroxyacetophenone under basic conditions (yield: 70%). Treatment with bromine in CH_3_Cl gave the desired chromene **2** (yield: 52%) which was oxidized by oxone in THF/H_2_O to provide 2-[4-(methylsulfonyl)phenyl]-4*H*-chromen-4-one **3** (yield: 78%). Chalcone **1** was converted to 3-hydroxy-2-[4-(methylsulfonyl)phenyl]-4*H*-chromen-4-one (**4**) (yield: 78%) by treatment with H_2_O_2_ in a hydro-alcoholic solution under basic conditions. Compound **4** was reacted with the appropriate alkyl halide to afford **5a–d** (yield: 31-40%). To obtain {2-[4-(methylsulfonyl)phenyl]-4-oxo-4*H*-chromen-3-yl}acetate **6**, compound **4** was treated with acetyl chloride in acetonitrile under basic conditions using TEA (yield: 68%). All compounds were pure and stable. The compounds were characterized by ^1^H and ^13^C nuclear magnetic resonance, infrared, mass spectrometry, and CHN analysis.

**Sch. 1:**
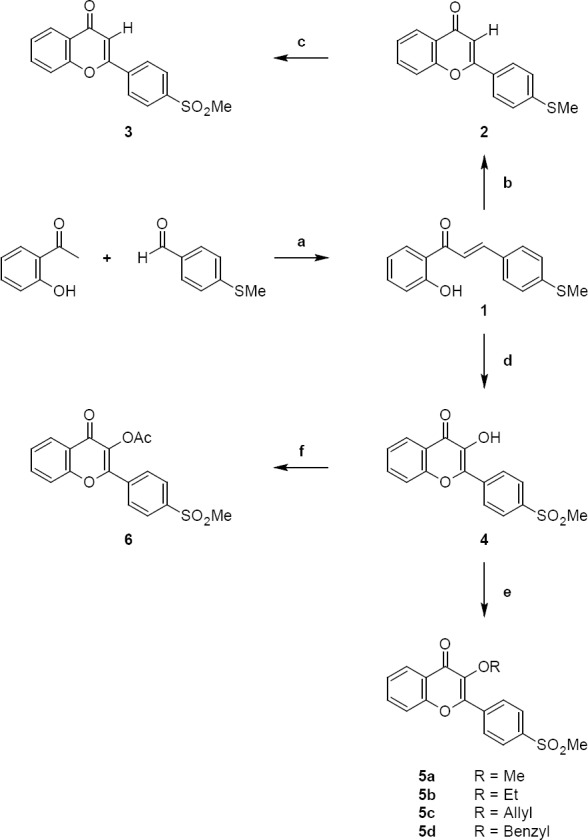
Reagents and conditions: (a) NaOH, MeOH, 25°C, 2 h; (b) Br_2_, CHCl_3_, RT; (c) Oxone, THF/H_2_O, 12 h; (d) H_2_O_2_ 30%, NaOH, EtOH; (e) Alkyl halide, NaOH, THF; (f) Acetyl chloride, TEA, ACN.

**Fig. 2:**
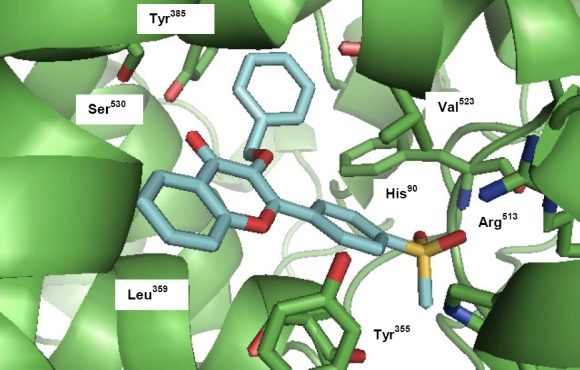
Docking 3-(Benzyloxy)-2-[4-(methylsulfonyl)phenyl]-4*H*-chromen-4-one (5d) in the active site of the murine COX-2 isozyme

The synthesized compounds **3–6** were evaluated for their ability to inhibit the COX-1 and COX-2 isozymes. *In vitro* structure-activity relationships acquired for this group of 2-[4-(methylsulfonyl)phenyl]-4*H*-chromen-4-one derivatives showed fairly good to potent inhibiting COX-2 activities, displaying IC_50_ values from 0.07 to 0.13 μM (see enzyme inhibition data in Table 1). According to these results, compound **5d** was the most potent (IC_50_ = 0.07 μM for COX-2) and selective (SI = 287.1) COX-2 inhibitor among the synthesized compounds. The obtained results indicated that the relative COX-2 potency and COX-2 selectivity profiles for the 4-*H*-chromen-4-one derivatives **3–6**, with respect to the substituent at the C-3 chromene moiety (R), were benzyl > acetyl > allyl > Et > Me > H > OH which revealed that the size and nature of the substituent at the C-3 chromene moiety can affect both COX-2 inhibitory activity and selectivity. Our results showed that the increase in lipophilic properties of substituents on the C-3 chromene ring increased COX-2 inhibitory potency and selectivity. Accordingly, the compound possessing the benzyloxy group on the C-3 chromene ring was the most selective COX-2 inhibitor compared with those having smaller groups. This effect may be explained by the steric parameters for interaction with the COX-1 active binding site. The orientation of the highly potent and selective COX-2 inhibitor, 3-(benzyloxy)-2-[4-(methylsulfonyl)phenyl]-4*H*-chromen-4-one, **5d** in the COX-2 active site, was examined by a docking experiment (Fig. 2) [19, 20]. Our molecular modeling study showed that the *p*-MeSO_2_-phenyl moiety was well-oriented towards the COX-2 active site. One of the *O*-atoms of the *p*-MeSO_2_ substituent formed a hydrogen bonding interaction with the amino group of Arg^513^ (distance = 4.0 Å), whereas the other *O*-atom was close to the other hydrogen of this amino acid (distance = 4.1 Å). Also, the distance between the *O*-atom of the C=*O* group attached to the chromene ring and O*H* of Ser^530^ was 3.7 Å, which can provide a hydrogen bonding interaction. On the other hand, the oxygen atom of the chromene ring was close to the O*H* group of Tyr^355^ (distance = 3.3 Å), and the phenyl ring of the benzoyl moiety was close to the hydrophobic side chain of Tyr^385^ and Val^523^. These data, together with experimental results, can explain the high potency and selectivity of compound **5d**.

**Tab. 1:**
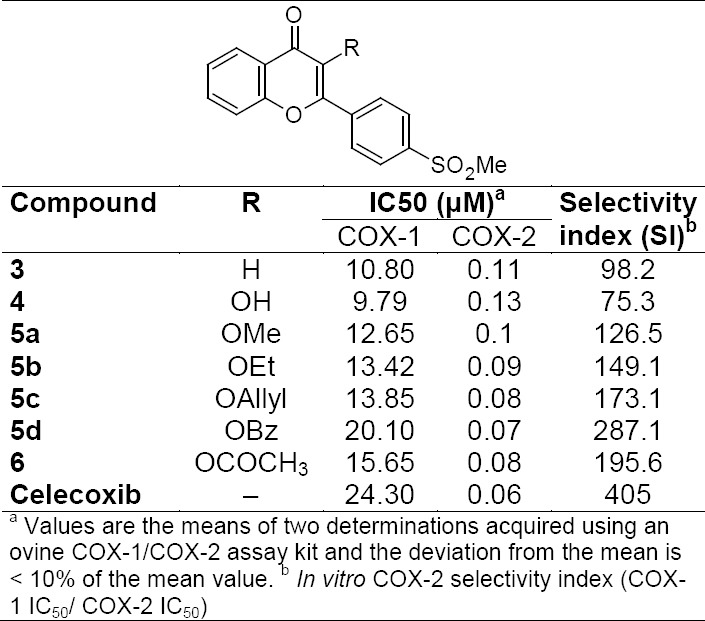
*In vitro* COX-1 and COX-2 enzyme inhibition data

## Conclusion

In conclusion, we designed and synthesized a series of new 2-[4-(methylsulfonyl)phenyl]-4*H*-chromen-4-ones. All the target compounds exhibited moderate-to-high COX-2 inhibitory activity. Our results also demonstrated that the nature and size of the substituent at position 3 of the chromene scaffold is important for COX-2 inhibitory activities.

## Experimental

### Chemistry

All chemicals, reagents, and solvents used in this study were purchased from Merck AG and Aldrich Chemical. Melting points were determined with a Thomas–Hoover capillary apparatus. Infrared spectra were acquired using a Perkin Elmer Model 1420 spectrometer. A Bruker FT-500 Plus 400 MHz instrument (Brucker Biosciences, USA) was used to acquire the ^1^HNMR and ^13^CNMR spectra with TMS as the internal standard. Chloroform-*d* and DMSO-*d*_6_ were used as solvents. Coupling constant (*J*) values are estimated in hertz (Hz) and spin multiples are given as s (singlet), d (double), t (triplet), q (quartet), m (multiplet), and br (broad). The mass spectral measurements were performed on the 6410 Agilent, low-resolution mass spectra were acquired with a MAT CH5/DF (Finnigan) mass spectrometer that was coupled online to a Data General DS 50 data system. Electron-impact ionization was performed at an ionizing energy of 70 eV with a source temperature of 250°C. The LCMS triple quadruple mass spectrometer (LCMS) was used with an electrospray ionization (ESI) interface. Microanalyses, determined for C, H, and N were within ± 0.4% of their theoretical values.

#### (2E)-1-(2-Hydroxyphenyl)-3-[4-(methylthio)phenyl]prop-2-en-1-one (1)

The mixture of 4-(methylthio)benzaldehyde (1 g, 6.5 mmol), 2-hydroxyacetophenone (0.8 g, 5.8 mmol), 40% w/v aqueous NaOH in ethanol was stirred at room temperature for 2 h. The mixture was cooled and poured into water to obtain the precipitate. The crude product was filtered off and recrystalized from MeOH to afford chalcone **1**.

Yield: 70%; mp: 86°C; IR (KBr): ν (cm^−1^) 1650 (C=O), 2780-3680 (OH); MS (*m/z*, %): 270.3 (M^+^, 80), 223.3 (45), 150.2 (90), 137.1 (100), 121.1 (80), 93 (45), 65 (70).

#### 2-[4-Methylthio)phenyl]-4H-chromen-4-one (2)

To the mixture of compound **1** (0.5 g, 1.8 mmol) dissolved in acetic acid was added bromine dropwise. The mixture was stirred at room temperature for 2 h. Then 1% w/v aqueous metabisulfite was added to produce the precipitate. The colorless solid was separated by filtration and the product was washed with water and dissolved in EtOH and stirred for 3 h under basic conditions. The progress of the reaction was followed by TLC. After the completion of the reaction, the obtained precipitate was filtered, washed with water, and then recrystalized from EtOH to afford chromene **2**.

Yield: 52%; mp: 105°C; IR (KBr): ν (cm^−1^) 1650 (C=O); ^1^HNMR (500 MHz, CDCl_3_): δ ppm 2.59 (s, 3H, CH_3_), 6.84 (s, 1H, =CH), 7.38 (d, 2H, 4-methylthiophenyl H_3_ & H_5_, *J* = 8.6 Hz), 7.45 (t, 1H, Phenyl H_6_), 7.60 (d, 1H, Phenyl H_8_, *J* = 8.3 Hz), 7.72 (t, 1H, Phenyl H_7_), 7.88 (d, 2H, 4-methylthiophenyl H_2_ & H_6_, *J* = 8.6 Hz), 8.26 (d, 1H, Phenyl H_5_, *J* = 7.9 Hz(; ^13^CNMR (300 MHz, CDCl_3_): 15.1, 105.1, 117.5, 123.6, 126.6, 126.7, 126.8, 127.1, 128.1, 131.2, 135.4, 142.5, 157.3, 163.8, 180.1; MS (*m/z, %*): 268.2 (M^+^, 90), 221.2 (65),148 (100), 120.1 (70), 89.1 (70), 60 (55). Anal. Calcd. for C_16_H_12_O_2_S: C, 71.62; H, 4.51. Found: C, 71.82; H, 4.72.

#### 2-[4-(Methylsulfonyl)phenyl]-4H-chromen-4-one (3)

One gram (3.7 mmol) of **2** was dissolved in 20 ml THF, and 6 g oxone in THF/water (1:1) was added. The mixture was stirred at room temperature overnight. THF was evaporated and the residue was extracted with chloroform. The organic phase was washed three times with saturated NaHCO_3_ and dried with anhydrous Na_2_SO_4_. Evaporation of the solvent under reduced pressure yielded a yellow crystalline solid which recrystalized in EtOH.

Yield: 78%; mp: 187°C; IR (KBr): ν (cm^−1^) 1150,1300 (SO_2_), 1650 (C=O); ^1^HNMR (500 MHz, CDCl_3_): δ ppm 3.16 (s, 3H, SO_2_CH_3_), 6.94 (s, 1H, =CH), 7.50 (t, 1H, Phenyl H_6_), 7.64 (d, 1H, Phenyl H_8_, *J* = 8.1 Hz), 7.78 (t, 1H, Phenyl H_7_), 8.14 (d, 2H, 4-methylsulfonylphenyl H_2_ & H_6_, *J* = 7.2 Hz), 8.17 (d, 2H, 4-methylsulfonylphenyl H_3_ &H_5_, *J* = 7.2 Hz), 8.29 (dd, 1H, Phenyl H_5_, *J* = 7.9 Hz, *J* = 1.6Hz); ^13^CNMR (300 MHz, CDCl_3_): 44.3, 109.2, 118.1, 123.7, 125.6, 125.6, 127.1, 128.1, 134.2, 136.7, 142.8, 156.1, 160.8, 177.9; MS(*m/z*,%): 300.1 (M^+^,70), 220 (45), 209 (40), 165 (30), 119.9 (95), 91.9 (100); Anal. Calcd. for C_16_H_12_O_4_S: C, 63.99; H, 4.03. Found: C, 64.12; H, 4.22.

#### 3-Hydroxy-2-[4-(methylsulfonyl)phenyl]-4H-chromen-4-one (4)

To the mixture of compound **1** (0.8 g, 3 mmol) dissolved in EtOH was added 30% w/v aqueous H_2_O_2_ (1 ml) and 5% w/v aqueous NaOH at 0–5°C. The mixture was stirred for 3 h at room temperature. After the completion of the reaction, the mixture was acidified by 2 M HCl and the precipitate was filtered and crystallized by EtOAc (**4**).

Yield: 78%; mp: 198–199°C; IR (KBr): ν (cm^−1^) 1150,1300 (SO_2_), 1640 (C=O), 2780-3340 (OH); ^1^HNMR (500 MHz, DMSO-*d_6_*): δ ppm 3.16 (s, 3H, SO_2_CH_3_), 5.38 (bs, 1H, OH), 7.49 (t, 1H, Phenyl H_6_), 7.67 (d, 1H, Phenyl H_8_, *J* = 8.4 Hz), 7.80 (t, 1H, Phenyl H_7_), 8.14 (d, 2H, 4-methylsulfonylphenyl H_2_ & H_6_, *J* = 7.0 Hz), 8.31 (dd, 1H, Phenyl H_5_, *J* = 7.0 Hz, *J* = 1.5 Hz), 8.51 (d, 2H, 4-methylsulfonylphenyl H_3_ & H_5_, *J* = 7.0 Hz); ^13^CNMR (300 MHz, DMSO-*d_6_*): 44.3, 116.1, 121.7, 123.4, 125.8, 127.4, 128.2, 135.2, 135.3, 136.5, 140.2, 146.9, 156.2, 172.8; MS (*m/z*, %): 316.3 (M^+^, 35), 191 (30), 147 (20), 56.9 (100); Anal. Calcd. for C_16_H_12_O_5_S: C, 60.75; H, 3.82. Found: C, 61.01; H, 4.08.

### General Procedure for the Synthesis of 3-alkoxy-2-[4-(methylsulfonyl)phenyl]-4H-chromen-4-ones (5a–d)

To a solution of compound **4** (1 g, 3.16 mmol) dissolved in THF was added the appropriate (15.8 mmol) alkyl halide, 10% w/v aqueous NaOH, and the resultant mixture was stirred at room temperature for 12 h. After the completion of the reaction, the solvent was evaporated and the residue was extracted with chloroform. The organic phase was washed three times with saturated NaHCO_3_ and dried over anhydrous Na_2_SO_4_. The collected organic phase was concentrated under vacuum and the obtained residue was crystallized by EtOH (yield: 31–40%).

#### 3-Methoxy-2-[4-(methylsulfonyl)phenyl]-4H-chromen-4-one (5a)

Yield: 38%; mp: 92–93°C; IR (KBr): ν (cm^−1^) 1145-1310 (SO_2_), 1650 (C=O); ^1^HNMR (500 MHz, CDCl_3_): δ ppm 3.11 (s, 3H, SO_2_CH_3_), 3.94 (s, 3H, OCH_3_), 7.41 (t, 1H, Phenyl H_6_), 7.54 (d, 1H, Phenyl H_8_, *J*= 8.4 Hz), 7.69 (t, 1H, Phenyl H_7_), 8.07 (d, 2H, 4-methylsulfonylphenyl H_2_ & H_6_, *J* = 8.3 Hz), 8.25 (d, 1H, Phenyl H_5_, *J* = 7.9 Hz), 8.29 (d, 2H, 4-methylsulfonylphenyl H_3_ & H_5_, *J* = 8.3 Hz); ^13^CNMR (300 MHz, CDCl_3_): 44.3, 60.2, 117.9, 123.9, 124.9, 125.7, 127.4, 129.2, 133.9, 135.9, 141.7, 142.3, 152.7, 155.1, 174.8; MS (m/z, %): 330.1 (M^+^, 35), 329.1 (100), 250 (45), 221.1 (25), 121.1 (35), 92.1 (15); Anal. Calcd. for C_17_H_14_O_5_S: C, 61.81; H, 4.27. Found: C, 61.99; H, 4.39.

#### 3-Ethoxy-2-[4-(methylsulfonyl)phenyl]-4H-chromen-4-one (5b)

Yield: 31%; mp: 137–138°C; IR (KBr): ν (cm^−1^) 1150,1300 (SO_2_), 1650 (C=O); ^1^HNMR (500 MHz, CDCl_3_): δ ppm 1.23 (s, 3H, CH_3_), 3.11(s, 3H, SO_2_CH_3_), 4.18 (m, 2H, OCH_2_), 7.40 (t, 1H, Phenyl H_6_), 7.53 (d, 1H, Phenyl H_8_, *J* = 8.45 Hz), 7.69 (t, 1H, Phenyl H_7_), 8.06 (d, 2H, 4-methylsulfonylphenyl H_2_ & H_6_, *J* = 8.2 Hz), 8.25 (d, 1H, Phenyl H_5_, *J* = 7.9 Hz), 8.33 (d, 2H, 4-methylsulfonylphenyl H_3_&H_5_, *J* = 8.2 Hz); ^13^CNMR (300 MHz, CDCl_3_) 15.6, 44.4, 68.7, 117.9, 124.0, 124.9, 125.8, 127.3, 129.4, 133.8, 136.3, 141.4, 141.6, 152.9, 155.1, 175.1; MS (m/z, %): 344.2 (M^+^, 100), 329.2 (55), 237.2 (60), 221.1 (60), 152.2 (60), 120.1 (55), 92.1 (40); Anal. Calcd. for C_18_H_16_O_5_S: C, 62.78; H, 4.68. Found: C, 62.99; H, 4.87.

#### 3-(Allyloxy)-2-[4-(methylsulfonyl)phenyl]-4H-chromen-4-one (5c)

Yield: 40%; mp: 116–118°C; IR (KBr): ν (cm^−1^) 1155,1300 (SO_2_), 1630 (C=O); ^1^HNMR (500 MHz, CDCl_3_): δ ppm 3.11 (s, 3H, SO_2_CH_3_), 4.70 (d, 2H, =CH_2_, *J* = 6.2 Hz), 5.16 (d, 1H, OCH, *J* = 9.6 Hz), 5.29 (d, 1H, OCH, *J* = 17.1Hz) 5.86 (m, 1H, C=CH), 7.41 (t, 1H, Phenyl H_6_), 7.53 (d, 1H, Phenyl H_8_, *J* = 8.4 Hz), 7.69 (t, 1H, Phenyl H_7_), 8.06 (d, 2H, 4-methylsulfonylphenyl H_2_ & H_6_, *J* = 8.5 Hz), 8.25 (d, 1H, Phenyl H_5_, *J* = 8.0 Hz), 8.31 (d, 2H, 4-methylsulfonylphenyl H_3_ & H_5_, *J* = 8.5 Hz); ^13^CNMR (300 MHz, CDCl_3_) 44.3, 73.4, 117.9, 119.2, 123.9, 124.9, 125.8, 127.3, 129.4, 132.9, 133.8, 136.1, 140.8, 141.7, 153.1, 155.1, 174.9; LC-MS (ESI) m/z: 357.30 (M+1); Anal. Calcd. for C_19_H_16_O_5_S: C, 64.03; H, 4.53. Found: C, 64.23; H, 4.78.

#### 3-(Benzyloxy)-2-[4-(methylsulfonyl)phenyl]-4H-chromen-4-one (5d)

Yield: 31%; mp: 140–142°C; IR (KBr): ν (cm^−1^) 1140,1300 (SO_2_), 1640 (C=O);^1^HNMR (500 MHz, CDCl_3_): δ ppm 3.14 (s, 3H, SO_2_CH_3_), 5.25 (s, 2H, OCH_2_), 7.27 (d, 2H, Benzyloxy H_2_ & H_6_, *J* = 7.5 Hz), 7.30 (m, 3H, Benzyloxy H_3-_H_5_), 7.48 (t, 1H, Phenyl H_6_), 7.57 (d, 1H, Phenyl H_8_, *J* = 8.4 Hz), 7.75 (t, 1H, Phenyl H_7_), 8.01 (d, 2H, 4-methylsulfonylphenyl H_2_ & H_6_, *J* = 8.5 Hz), 8.18 (d, 2H, 4-methylsulfonylphenyl H_3_ & H_5_, *J* = 8.5 Hz), 8.34 (d, 1H, Phenyl H_5_, *J* = 8.0 Hz); ^13^CNMR (300 MHz, CDCl_3_): 44.2, 74.2, 117.9, 123.9, 124.9, 125.6, 127.0, 128.2, 128.3, 128.9, 129.5, 133.8, 135.8, 135.9, 140.4, 141.5, 153.7, 155.0, 174.8; MS (m/z, %): 406 (M^+^, 5), 391.8 (90), 311.9 (45), 164.8 (60), 91(100); Anal. Calcd. for C_23_H_18_O_5_S: C, 67.97; H, 4.46. Found: C, 67.79; H, 4.59.

#### 2-[4-(Methylsulfonyl)phenyl]-4-oxo-4H-chromen-3-yl acetate (6)

To a cooled down solution of compound **3** dissolved in ACN was added acetyl chloride slowly under basic conditions using TEA. The mixture was stirred at room temperature for 3 h. After evaporation of the solvent, the residue was dissolved in CH_2_Cl_2_ and washed twice with saturated NaHCO_3_ and dried over anhydrous Na_2_SO_4_ and concentrated under vacuum to obtain the residue, which was purified by flash chromatography (*n*-hexan/EtOAc=1/20), and gave yellow crystalline solid (**6**).

Yield: 68%; mp: 175°C; IR (KBr): ν (cm^−1^) 1150,1310 (SO_2_), 1645(C=O), 1760 (COCH_3_); ^1^HNMR (500 MHz, CDCl_3_): δ ppm 2.42 (s, 3H, COCH_3_), 3.17 (s, 3H, SO_2_CH_3_), 7.51(t, 1H, Phenyl H_6_), 7.61 (d, 1H, Phenyl H_8_, *J* = 8.4 Hz), 7.79 (t, 1H, Phenyl H_7_), 8.06 (d, 2H, 4-methylsulfonylphenyl H_2_ & H_6_, *J* = 8.5 Hz), 8.12 (d, 2H, 4-methylsulfonylphenyl H_3_ & H_5_, *J* = 8.5 Hz), 8.31 (d, 1H, Phenyl H_5_, *J* = 7.9 Hz); ^13^CNMR (300 MHz, CDCl_3_): 20.5, 44.2, 118.0, 123.4, 125.5, 126.1, 127.6, 129.1, 134.3, 134.5, 135.0, 142.5, 153.8, 155.4, 167.7, 171.8; LC-MS (ESI) m/z: 359.40 (M+1); Anal. Calcd. for C_18_H_14_O_6_S: C, 60.33; H, 3.94. Found: C, 60.59; H, 4.14.

## Docking Studies

In order to perform the docking studies, AutodockVina software Version 1-0-3-win32 was used. The X-ray crystal structure of the selective COX-2 in the complex with SC-558 (entry code 6COX) was obtained from the RCSB Protein Data Bank (http://www.rcsb.org/pdb/home/home.do). The ligand molecules were constructed using the hyperchem release 8 and subsequently minimized. The protein structure was prepared for docking using the AUTODOCK Tool. Polar hydrogens were added, nonpolar hydrogens were merged, and then the Kallman united atom charge and atom type parameter were added to 6COX. Grid map dimensions (20×20×20) were set surrounding the active site. The energy-minimized ligands were docked on SC-558 in the PDB file 6COX after the co-crystallized ligand and all water molecules were removed. The quality of the docked structures was assessed by measuring the intermolecular energy of the ligand-enzyme assembly. The purpose of docking is to search for favorable binding configurations between the small flexible ligands and the rigid protein to explain the selectivity pattern acquired in biological assays at the molecular level.

## Biological Assay

The ability of the test compounds listed in Table 1 to inhibit ovine COX-1 and COX-2 (IC_50_ value, μM) was determined using the chemiluminescent enzyme assay kit (Cayman Chemical, Ann Arbor, MI, USA) according to our previously reported method [21].
